# Austrian consensus guidelines on imaging requirements prior to hepatic surgery and during follow-up in patients with malignant hepatic lesions

**DOI:** 10.1007/s00508-018-1387-z

**Published:** 2018-08-30

**Authors:** Dietmar Tamandl, Ahmed Ba-Ssalamah, Gernot Böhm, Klaus Emmanuel, Rosemarie Forstner, Reinhold Függer, Benjamin Henninger, Oliver Koch, Claus Kölblinger, Hans-Jörg Mischinger, Wolfgang Schima, Helmut Schöllnast, Stefan Stättner, Klaus Kaczirek

**Affiliations:** 1 Department of Biomedical Imaging and Image-Guided Therapy, Medical University of Vienna, Währinger Gürtel 18–20, 1090 Vienna, Austria; 2 Department of Surgery, Medical University of Vienna, Währinger Gürtel 18–20, 1090 Vienna, Austria; 3 Department of Radiology, Ordensklinikum Linz, Linz, Austria; 4 Department of Surgery, Uniklinikum Salzburg, Paracelsus Private Medical University, Salzburg, Austria; 5 Department of Radiology, Uniklinikum Salzburg, Paracelsus Private Medical University, Salzburg, Austria; 6 Department of Surgery, Ordensklinikum Linz, Linz, Austria; 7 Department of Radiology, Medical University of Innsbruck, Innsbruck, Austria; 8 Department of Radiology, Hospital Barmherzige Schwestern Ried, Ried im Innkreis, Austria; 9 Department of Surgery, Medical University of Graz, Graz, Austria; 10 Department of Radiology, Göttlicher Heiland Hospital, Barmherzige Schwestern Hospital and Sankt Josef Hospital, Vinzenzgruppe, Vienna, Austria; 11 Department of Radiology, Medical University of Graz, Graz, Austria; 12 Department of Surgery, Medical University of Innsbruck, Innsbruck, Austria

**Keywords:** Imaging, Hepatic surgery, Colorectal liver metastases, Hepatocellular carcinoma, Cholangiocarcinoma

## Abstract

Rapid advances in imaging technology have improved the detection, characterization and staging of colorectal liver metastases, hepatocellular carcinoma and cholangiocarcinoma. A variety of imaging modalities are available and play a pivotal role in the work-up of patients, particularly as imaging findings determine resectability. Surgery often represents the only measure that can render long-term survival possible. Imaging is also indispensable for the assessment of responses to neoadjuvant treatment and for the detection of recurrence. At a consensus meeting held in June 2017 in Vienna, Austria, Austrian experts in the fields of surgery and radiology discussed imaging requirements prior to and after hepatic surgery for malignant liver lesions. This consensus was refined by online voting on a total of 47 items. Generally, the degree of consensus was high. The recommendations relate to the type of preferred preoperative imaging modalities, technical settings with respect to computed tomography and magnetic resonance imaging, use of contrast agents, reporting, postoperative follow-up, and long-term follow-up. Taking local resources into account, these consensus recommendations can be implemented in daily clinical practice at specialized centers as well as outpatient diagnostic institutes in Austria.

## Introduction

Cancerous diseases of the liver can occur due to primary lesions, such as hepatic cellular carcinoma (HCC) and cholangiocarcinoma (CCC) or metastatic lesions. In the majority of cases (95%) liver lesions are of metastatic origin [1] with colorectal carcinoma representing the most common source (colorectal liver metastases, CLM). In Austria, there are approximately 4500 new cases of colorectal cancer each year, with about 30–50% presenting with or developing CLM, as was assessed by “Statistik Austria” in 2015. For HCC and CCC combined, the yearly incidence in 2015 was 940 cases, with approximately 20–30% being potentially resectable.

Hepatic surgery plays a vital role in patient management in all three settings. For CLM it has been shown that resection improves the prognosis of patients and is the only treatment associated with long-term survival in patients with liver-limited disease [2–6]. Likewise, patients with early-stage HCC can be offered a potentially curative approach if the tumors are found to be resectable [7]. In patients with CCC, who generally have a poor prognosis, surgical resection again represents the only potential strategy to permanently eradicate the disease [8–10].

Preoperative imaging is crucial for the detection, characterization and staging of hepatic lesions. Decisions concerning patient management and the appraisal of patient outcome require determination of the exact extent of the disease. Nowadays, a multitude of therapeutic options including surgery, systemic treatment and locoregional therapies are available for malignant liver disease and treatment choices in every individual case depend on diagnostic imaging to a considerable degree. Cases that are (potentially) eligible for resection need to be distinguished from those where surgical intervention is unlikely to be successful, as incomplete resection does not prolong survival. Imaging is also vital for the assessment of treatment response and for the detection of recurrence during follow-up. At the same time, radiologists can choose among a range of imaging techniques, including contrast-enhanced computed tomography (CT), magnetic resonance imaging (MRI), magnetic resonance cholangiopancreatography (MRCP), positron emission tomography-CT (PET-CT), ultrasonography (US) and in selected cases, angiography. Functional techniques, such as perfusion CT-MRI and diffusion-weighted MRI and PET/MRI provide additional information related to tumor biology.

As the array of preoperative imaging has expanded over the past years, recommendations detailing the appropriate use of various techniques in different settings appear necessary. In addition, patient characteristics, such as steatosis or previous treatments (e. g., neoadjuvant chemotherapy) can affect imaging, which further increases the complexity of pretherapeutic decision making; however, no international guidelines have been published on preoperative imaging in the setting of hepatic surgery to date, although there are a large number of recent reviews and meta-analyses investigating radiologic evaluation in CLM [11–13], HCC [14–21] and CCC [22–24].

The purpose of this consensus statement is to establish recommendations for imaging requirements prior to hepatic surgery and during follow-up in patients with liver malignancies. These recommendations take local requirements and resources into account and were designed to be applicable both for diagnostic imaging in outpatient centers as well as in tertiary referral centers.

## Material and methods

For establishing consensus, a multi-step modified Delphi technique was used [25].

### Step 1

Of the organizing members two (D. T. and K. K.) gathered and reviewed the current literature dealing with imaging in the setting of liver resections for malignant hepatic tumors. Due to advances in the field, only reports published in the last 15 years were considered relevant for this topic. The aim was to establish a consensus regarding imaging for CLM, HCC and CCC, since these represent the most common indications for a liver resection. A catalogue of 27 items was established that covered the most important topics in this field, grouped by various clinical areas of interest.

### Step 2

At an expert meeting conducted on 27 June 2017 in Vienna, Austria, 13 Austrian experts in the fields of gastrointestinal radiology and liver surgery were invited to participate in the first open discussion round table and 11 members were present during this first panel meeting. The meeting was moderated by two panel members (K. K. and D. T.) who kept track of the statements and additional comments made. All items were discussed and additional topics were included if requested by the majority of panelists. If required, an ad hoc review of the pertinent topic using a PubMed search was performed to clarify open questions. After this meeting, a list of 47 items was generated, which already included a selective approach for each entity, CLM, HCC and CCC.

### Step 3

For online voting, experts scored each item on a scale from 1 to 5, with 1 representing no agreement and 5 representing full agreement. The scores obtained for each item were added together, which resulted in minimum and maximum scores of 13 and 65, respectively. In addition, the degree of consensus obtained for each item was calculated in percent. Participants could elect to perform the voting anonymously. A total of 47 items were put to the vote, as mentioned above. Where applicable, the recommendations were rated separately for CLM, HCC and CCC. These attributions are discerned in the table by the use of the letters A, B and C, with A denoting CLM, B denoting HCC, and C denoting CCC.

### Step 4

All voting results were collected and it was decided whether consensus (≥80% agreement) had been obtained. Comments made by individual panelists were noted and were included in the discussion of this manuscript.

### Step 5

In the last step, after setting up the manuscript, each panel member was able to make additional comments on individual topics. Of note, if an item had already reached consensus in Step 3 and 4, no alterations to this item were possible at this stage.

The recommendations should be valid for any patient requiring imaging work-up before liver resection for malignancy and during follow-up. Certainly, specific circumstances, such as allergy to contrast agents or contraindications against MRI have to be considered individually by the treating physician.

The recommendations were created in accordance with the AGREE II tool [26], which can be used in the quality-assessment of guidelines and consensus statements. All items have been successfully verified to be fulfilled in the presented version of this manuscript.

## Results

The items were summarized in 18 groups ranging from general requirements in the preoperative setting to reporting and follow-up (Tables 1 and 2). A high degree of consensus was obtained for the majority of items (mean consensus rating 95%, range 65–100%).

**Table 1:** Recommendations and degree of consensus, part 1

Item no.	Statement	Consensus level, %
**General preoperative requirements, computed tomography** **If not specified further, recommendation relates to all entities**		
*Which imaging modalities should be used for the workup of a liver lesion before resection?*		
1_CLM_	CT of the chest and abdomen should be performed as basic examinations. Negative results preclude further assessment. Contrast-enhanced MRI is only indicated in (potentially) resectable lesions	100
1_CLM_i	If CT reveals visible steatosis, which implicates the risk of false negative findings, additional MRI should be considered, even if CT is negative	88
1_CLM_ii	In lean patients, sonography is optional as an additional assessment	65
1_HCC_	Lesions of uncertain malignancy suspected in patients at risk for HCC: CT serves as the basic examination with the purpose of characterizing the lesions. If locoregional techniques are feasible: – Contrast-enhanced MRI (number of lesions, hepatic function, extent of disease), particularly if no cirrhosis is present, and – CT of the chest (extent of extrahepatic disease)	100
1_CCC_	CT is the basic examination; further characterization calls for contrast-enhanced MRI plus MRCP sequences. It is strongly recommended to perform MRCP sequences prior to stenting	93
*Is CT of the chest and abdomen always mandatory for preoperative assessment?*		
2	In all three entities, CT of the chest and abdomen is mandatory	98
2_HCC_i	An exception is the patient at risk who presents with a hepatic lesion (suspected HCC in liver cirrhosis, chronic viral hepatitis, or steatohepatitis). Here, CT assessment of the entire abdomen and chest should only be performed if a lesion suggesting HCC is present	100
*Which series are advisable for abdominal/liver CT?*		
3_CLM_	For the initial assessment, we strongly recommend arterial and portal venous phases. Additional unenhanced series prior to the application of contrast agents are optional	97
3_HCC_	3-phasic or 4‑phasic with arterial/portal venous/equilibrium phases, with the unenhanced phase being optional	97
3_CCC_	3-phasic or 4‑phasic with arterial/portal venous/equilibrium phases, with the unenhanced phase being optional	95
*Which slice thickness should be used for reconstruction of axial series?*		
4	The maximum slice thickness should not exceed 3 mm in all entities	98
4i	For the assessment of arterial vessels, a maximum slice thickness of 1 mm is optional in case of specific surgical issues or in patients with Klatskin tumors	88
*Which types of reconstruction are recommended?*		
5	Coronal reconstruction for portal venous and arterial phases, with a slice thickness of 3 mm. Sagittal reconstructions for spine assessment	95
*Which amount of contrast agent should be used for CT?*		
6	An iodine dose of 0.6 g/kg body weight (i. e. 2 ml/kg for an agent with an iodine content of 300 mg/ml) should be used for the standard 64 slice system. Reductions might be possible when using modern generation scanners	95
*Flow rate and timing of contrast agent application*		
7_CLM_	3–5 ml/s	88
7_HCC_	4 ml/s	82
7_HCC_i	Imaging of the arterial phase should focus at the late arterial phase, i. e. using bolus tracking with a delay of 15–18 s. Definition of the late arterial phase: enhancement of the hepatic artery and portal vein	98
7_CCC_	3–5 ml/s	87
*Reconstruction kernel, other scanner set-ups*		
8	Soft-tissue kernel according to the recommendations of the manufacturer	92
*Role of PET-CT*		
9	No role in the routine preoperative staging. PET-CT is a problem-solving tool in high-risk patients and should be used to investigate uncertain extrahepatic findings	97

Minimum voting score, 13; maximum voting score, 65, numbers are given as percentage of maximum voting score. Item no. refers to consecutive numbers of statements as discussed during the expert panel meeting. If a statement was made for one specific entity, this was specified with subscripts (CLM, HCC or CCC)

*PET-CT* positron emission tomography-computed tomography, *CT* computed tomography, *MRI* magnetic resonance imaging, *PET* positron emission tomography, *MRCP* magnetic resonance cholangiopancreatography, *CLM* colorectal liver metastases, *HCC* hepatic cellular carcinoma, *CCC* cholangiocellular carcinoma, *ADC* apparent diffusion coefficient, *Gd* gadolinium, *RECIST* Response Evaluation Criteria in Solid Tumors, *SOS* sinusoidal obstruction syndrome

**Table 2:** Recommendations and degree of consensus, part 2

Item no.	Statement	Consensus Level, %
**General preoperative requirements, magnetic resonance imaging** **If not specified further, recommendation relates to all entities**		
*Which field strength is required for liver MRI?*		
10	At least 1.5 T	100
*Which sequences should be performed as minimum requirements?*		
11	Axial T2w single-shot TSE, T2w-TSE (at least one T2w sequence with fat suppression), 2D/3D-T1w GRE with chemical shift imaging (in-phase and opposed-phase), dynamic 3D-T1w GRE with fat saturation dynamically after application of contrast agent (late arterial, portal venous, equilibrium phases; when using hepatocyte-specific contrast agents, hepatobiliary phase after 20 min or 60–120 min, depending on the contrast agent)	97
12	Diffusion-weighted sequences with low, intermediate and high b values of e. g. 50, 400, 800. ADC map	95
*MRI optimization*		
13	T2w-weighted sequences and DWI can be performed after the application of a contrast agent and T2w MRCP prior to the application of a contrast agent	92
14	In patients with significant ascites, paracentesis prior to imaging might be considered to improve image quality	82
*Which slice thicknesses should be used for MRI?*		
15	A maximum of 5 mm for 2D sequences, a maximum of 3 mm for 3D sequences	100
*Contrast agent: dosing, flow rate, bolus triggering*		
16	Dosing of contrast agents: 0.1 mmol/kg body weight for non-specific Gd chelate and 0.025 mmol/kg body weight for gadoxetic acid	100
17	Flow rate: contrast agents should be applied manually or by means of a contrast agent injector at a dose of 1 ml/s, followed by a sodium chloride flush	95
18	Bolus triggering for the arterial phase	97
*Which contrast agent for which entity?*		
19_CLM_	Gadoxetic acid for all patients	93
19_HCC_	Gd-containing contrast agent	82
19_CCC_	Peripheral CCC: gadoxetic acid; Klatskin tumor: Gd-containing contrast agent	85
19_CLM-CCC_i	Imaging studies for purposes of comparison should always be conducted using identical parameters. An exception to this rule is the arterial phase during follow-up of CLM, which can be dispensable	98
19_CLM_ii	In the setting of neoadjuvant chemotherapy of CLM, it is strongly recommended to conduct MRI before and after treatment or prior to surgery	92
**Information to be included in the report**		
20_CLM_i	Detailed description of metastatic lesions. Minimum requirements include the number of lesions, size (in mm) of the largest metastases and description of segmental distribution. Importantly, unaffected segments should be mentioned, as this allows for surgery to be considered or precluded in the first place. The term “multiple” should not be rashly used when lesions are countable. Differentiation from lesions that are reliably benign	100
20_CLM_ii	Proximity of lesions to vital structures (e. g., blood vessels, bile ducts). Presumed preservability of inflow/outflow	100
20_CLM_iii	Anatomical description, description of relevant normal variations	100
20_CLM_iv	If applicable, description of the quality of the parenchyma (e. g., cirrhosis, steatosis, etc.)	100
20_CLM_v	Description of extrahepatic lesions	100
20_HCC_	Number of unequivocal HCC lesions. The recommendations given for 20_CLM_i to v apply here as well, with a particular focus on portal vein thrombosis, if applicable	100
21_CLM_i	Response to prior neoadjuvant therapy and size of lesion(s; not necessarily according to RECIST)	100
21_CLM_ii	Metastatic lesions that have disappeared during neoadjuvant treatment should be mentioned	100
21_CLM_iii	Signs of chemotherapy-induced liver injury (e. g., steatosis, SOS)	100
**Postoperative follow-up (management of complications)**		
22	Postoperative CT using the arterial and portal venous phases is strongly recommended. For suspected bleeding, an unenhanced CT phase should also be performed	100
23	MR, MRCP in case of biliary complications	100
**Long-term follow-up**		
24	3–6 month intervals according to local preference	97
25	PET/CT should not be performed routinely, only in cases of unclear findings	97

Minimum voting score, 13; maximum voting score, 65, numbers are given as percentage of maximum voting score. Item no. refers to consecutive numbers of statements as discussed during the expert panel meeting. If a statement was made for one specific entity, this was specified with subscripts (CLM, HCC or CCC)

*CT* computed tomography, *MRI* magnetic resonance imaging, *PET* positron emission tomography, *MRCP* magnetic resonance cholangiopancreatography, *CLM* colorectal liver metastases, *HCC* hepatic cellular carcinoma, *CCC* cholangiocellular carcinoma, *ADC* apparent diffusion coefficient, *Gd* gadolinium, *RECIST* response evaluation criteria in solid tumors, *SOS* sinusoidal obstruction syndrome

### Preoperative imaging modalities and technical aspects pertaining to CT

With respect to imaging modalities to be used before surgery, the panel agreed that thoracic and abdominal CT constitutes the basic assessment method for all three entities. In cases of severe steatosis visible on CT, additional MRI might be considered even if no lesions are visible on CT. No consensus was obtained, whether sonography should be included in the initial assessment in lean patients; however, MRI should be definitely used in patients with resectable or potentially resectable CLM and in patients with HCC for whom locoregional techniques like resection or ablation appear feasible. In patients with CCC, contrast-enhanced MRI including MRCP sequences was recommended for further characterization of the lesions and should be performed prior to biliary stent placement. There was strong consensus among the panelists, that in all three tumor entities, the chest should be included in the preoperative staging CT. An exemption should be made in high-risk patients (e. g. patients with cirrhosis, chronic viral hepatitis or steatohepatitis) assessed for a liver lesion. In this case, a CT of the entire abdomen and chest should only be included if a lesion suspicious for HCC was detected in the liver (100% consensus).

The panelists strongly agreed (97% consensus) that an arterial and portal venous phase is recommended for the initial CT examination in patients with CLM. The HCC and CCC assessments require at least 3‑phasic scans with arterial/portal-venous/equilibrium phases, although there was no consensus on the ideal timing for the equilibrium phase, ranging from 3 to 5 min. Non-contrast scans are considered optional in all three entities. The slice thickness for reconstruction of the axial series should not exceed 3 mm in all entities. Thin-slice reconstructions with 1 mm especially for visualization of the arterial anatomy were considered optional in certain surgical situations and in patients with perihilar cholangiocarcinoma (Klatskin tumors). Coronal reconstructions were recommended for portal venous and arterial imaging, and sagittal reconstructions for spine assessment. With respect to the amount of contrast agent used for standard 64-slice CT, the panel recommended an iodine dose of 0.6 g/kg body weight. Dose reductions appear possible if modern generation scanners are used but have to be considered on an individual basis. Contrast material flow rates should be high, in the range of 3–5 ml/s for CLM and CCC, and 4 ml/s for HCC. In patients with HCC, a late arterial phase should be employed using bolus tracking. The panel agreed that PET/CT or PET/MRI has no role in the routine preoperative staging and should only be used as a problem solving tool in high-risk patients (e. g. with high tumor burden) as well as for the assessment of uncertain extrahepatic findings in CLM.

### MRI: technical aspects

For all aspects of liver MRI, the panel endorsed the recent recommendations made by the European Society of Gastrointestinal and Abdominal Radiology [26], with only slight modifications with respect to the preoperative setting. The panel agreed that a minimum field strength of 1.5 T is required for liver MRI. Mandatory MRI sequences include axial single-shot T2w-turbo spin echo (TSE) and intermediate T2w-TSE, at least one T2w sequence with fat suppression. Furthermore, 2D/3D-T1w gradient echo (GRE) with chemical shift imaging (in-phase and opposed-phase), and dynamic 3D-T1w GRE with fat saturation after administration of the contrast material should be applied. If hepatocyte-specific contrast agents are used, a hepatobiliary phase after 20 min (gadoxetic acid or Gd-EOB-DTPA, Bayer Healthcare [Berlin, Germany]) or 60–120 min (gadobenate dimeglumine, Bracco [Milan, Italy]), is recommended. Diffusion-weighted sequences (DWI) should be conducted with low, intermediate and high b‑values, e. g. 50, 400 and 800s/mm^2^, according to the manufacturer and field strength.

A MRI protocol streamlining is possible by performing T2w-sequences and DWI after contrast application and T2w MRCP beforehand. In patients with considerable ascites, paracentesis prior to imaging should be considered in order to improve image quality. The required slice thickness for MRI was unanimously agreed on with 5 mm for 2D sequences and a maximum of 3 mm for 3D sequences.

### Use of contrast agents for liver MRI

The experts generally agreed that non-specific gadolinium (Gd) chelates should be used at a dose of 0.1 mmol/kg body weight, while the recommended dose for the hepatocyte-specific agent gadoxetic acid is 0.025 mmol/kg body weight. Concerning flow rate, it is recommended to apply contrast agents manually or by means of an injector at a dose of 1 ml/s, followed by a sodium chloride flush. Bolus triggering is recommended for the arterial phase.

While the proposal to use gadoxetic acid in all patients with CLM was clearly supported by the majority of the panel (93% consensus), no consensus was obtained for the use of this contrast agent in HCC. Instead, it is recommended to apply any Gd-containing agent for the assessment of HCC (82% consensus). For CCC, the panel favored gadoxetic acid in patients with peripheral CCC, whereas any Gd-containing contrast agent should be used in the work-up of perihilar CCC (85% consensus). In general, the panelists noted that imaging studies should always be conducted using identical parameters for the purpose of comparison. In patients receiving neoadjuvant chemotherapy for CLM, it is strongly recommended to perform gadoxetic acid-enhanced MRI both before and after systemic treatment and prior to surgery.

### Reporting

A 100% degree of consensus was achieved concerning the necessary information to be included in the report. Almost all of the recommendations relate to CLM findings. The panel concluded that a detailed description of metastatic lesions, with minimum requirements including the number of lesions, the size of the largest lesions and the description of their segmental distribution should be mandatory. Importantly, unaffected segments should be mentioned, as this enables surgeons to consider or preclude resection up front. Other notable items refer to proximity of lesions to vital inflow/outflow structures, presumed preservability and the quality of the parenchyma, since the focus of technical resectability relies on the future liver remnant. Any observed anatomical variant needs to be noted in the report. In the context of neoadjuvant therapy, it is important to describe the response to treatment, which includes reporting lesions that have disappeared after chemotherapy and the description of signs of chemotherapy-induced liver injury. Size measurements should be included in the report to enable assessment of treatment response.

For HCC, the experts agreed that the number and distribution of unequivocal lesions, as well as parenchyma quality should be stated in the report. Portal or hepatic vein thrombosis as a marker of vascular invasion distinctly needs to be reported as well.

### Postoperative imaging studies (assessment of post-procedural complications)

The recommendations on postoperative follow-up received the maximum degree of consensus. A CT assessment using the arterial and portal venous phases was strongly recommended. A non-contrast phase should be added in cases of suspected bleeding. Both MRI and MRCP were recommended for the work-up of suspected biliary complications.

### Long-term follow-up

For long-term follow-up most of the experts favored 3‑6 month intervals according to local preference. In general, the panelists noted that imaging studies should always be conducted using identical parameters for the purpose of comparison. An exception to this rule is the arterial phase during follow-up, which might be dispensable for CT examinations in patients with CLM, which is known to seed hypovascular liver metastases. A PET/CT should be performed in patients presenting with equivocal findings and was not recommended as a routine imaging examination after liver resection.

## Discussion

The recommendations detailed here are based on a consensus that was achieved among Austrian hepatobiliary surgeons and gastrointestinal radiologists from four university hospitals and specialized high-volume liver centers, which was sought due to the lack of international guidelines on perioperative imaging in oncologic liver surgery. Taking local requirements into account, the consensus panel has strived to provide comprehensive recommendations covering both technical and clinical issues including reporting and the use of contrast agents. The recommendations given in this aticle apply to the first imaging examination performed at an outpatient imaging center up to the presurgical and postsurgical scans performed in tertiary referral centers. It should help clinicians to be provided with state-of-the art imaging for treatment planning, aiming to reduce the need for repeat scans in the inhospital setting, due to technical or methodological shortcomings. This in not only a task for the specialized abdominal radiologist, but also for general radiologists, who are encountered with liver lesions in every day’s practice.

A high level of agreement was achieved for most of the items, which reflects the clinical utility of the suggestions made here. All panel members are either working as active hepatobiliary surgeons or are active in the field of gastrointestinal radiology, with regular appearances on national and international conferences. Thus, the high degree of consensus might also reflect the regular exposure to this topic, which might not be the case for other general surgeons or radiologists.

There was in general an excellent (>80%) consensus on items dealing with CT imaging. For item 1C it was additionally noted by 2 experts, that they would include MRCP in suspected bile duct tumors only if there was evident bile duct dilatation on CT. There was no consensus, whether the equilibrium phase for CT imaging in hilar cholangiocarcinoma should be performed after 3 or 5 min, hence the exact time delay for the equilibrium phase was not included in the consensus table. For MRI assessment of liver tumors, a recent detailed consensus statement exists published by the European Society for Gastrointestinal and Abdominal Radiology (ESGAR; [26]), which was adapted to the requirements for liver resections. There was clear consensus about the use of gadoxetic acid in the presurgical staging of liver metastases (93%); Apart from radiological benefits of this methods, it has been showed to be cost-efficient if used in the preoperative workup of patients scheduled for surgery for CLM [27]. However, for HCC and CCC this contrast agent was not unequivocally recommended by all experts, hence for HCC any Gd-containing contrast agent was recommended and for CCC, gadoxetic acid was only considered for peripheral CCC.

There was a discussion about the dilution and injection rate of gadoxetic acid, due to the likelihood of significant respiratory artefacts in the arterial phase. One panel member recommended dilution of gadoxetic acid with respect to a recent publication that demonstrated decrease in respiratory artefacts [28]; however, this was not unequivocally agreed on, and noted that this reflects an off-label use, which can increase the contamination risk. In general, the injection rate recommended by the vendor should be followed; however, there was good consensus that in general 1 ml/s is recommendable.

There was only a moderate consensus whether ascites drainage should be performed prior to MRI imaging at 3.0 T, mainly due to work-flow related issues.

With respect to follow-up, it was noted by some panel members that PET/CT in general is a very useful tool for the follow-up of metastatic colorectal cancer, but for the particular assessment of the extent of liver involvement as well as for early detection of liver metastases it was deemed suboptimal. This was amended in the final statement.

In summary, these recommendations should guide the improvement of the diagnosis, characterization and staging of hepatic lesions in clinical practice, subsequently contributing to optimizing patient outcomes. This will help to avoid futile interventions in patients who are unlikely to benefit from surgery.
